# A First Step toward the Understanding of Implicit Learning of Hazard Anticipation in Inexperienced Road Users Through a Moped-Riding Simulator

**DOI:** 10.3389/fpsyg.2017.00768

**Published:** 2017-05-11

**Authors:** Mariaelena Tagliabue, Evelyn Gianfranchi, Michela Sarlo

**Affiliations:** Department of General Psychology, University of Padua,Padua, Italy

**Keywords:** hazard perception, electrodermal activity, moped-riding abilities, learning processes, novice road users

## Abstract

Hazard perception is considered one of the most important abilities in road safety. Several efforts have been devoted to investigating how it improves with experience and can be trained. Recently, research has focused on the implicit aspects of hazard detection, reaction, and anticipation. In the present study, we attempted to understand how the ability to anticipate hazards develops during training with a moped-riding simulator: the Honda Riding Trainer (HRT). Several studies have already validated the HRT as a tool to enhance adolescents’ hazard perception and riding abilities. In the present study, as an index of hazard anticipation, we used skin conductance response (SCR), which has been demonstrated to be linked to affective/implicit appraisal of risk. We administered to a group of inexperienced road users five road courses two times a week apart. In each course, participants had to deal with eight hazard scenes (except one course that included only seven hazard scenes). Participants had to ride along the HRT courses, facing the potentially hazardous situations, following traffic rules, and trying to avoid accidents. During the task, we measured SCR and monitored driving performance. The main results show that learning to ride the simulator leads to both a reduction in the number of accidents and anticipation of the somatic response related to hazard detection, as proven by the reduction of SCR onset recorded in the second session. The finding that the SCR signaling the impending hazard appears earlier when the already encountered hazard situations are faced anew suggests that training with the simulator acts on the somatic activation associated with the experience of risky situations, improving its effectiveness in detecting hazards in advance so as to avoid accidents. This represents the starting point for future investigations into the process of generalization of learning acquired in new virtual situations and in real-road situations.

## Introduction

Hazard perception is defined as the ability to predict in advance the potential occurrence of a dangerous event so as to behave in such a way to reduce the likelihood that an effective risk occurs. It is the first step of a series of processes that, among other higher-order cognitive abilities, is considered crucial in road safety (Horswill, 2016) and represents one of the experience-related elements that strongly contribute to the involvement of novice riders in on-road crashes (Haworth et al., 2005a). This is why in some countries, such as the UK and Australia, licensing programs include hazard-perception training, essentially characterized by showing potentially risky situations and asking the trainee to detect hazards (Haworth et al., 2005a). These kinds of training (which we will call “passive training methods” hereafter) are principally based on the reaction-time (RT) paradigm, in that participants’ RTs during the passive viewing of hazardous video clips are considered measures of efficiency in hazard perception and responding (Lim et al., 2014). Lim et al. (2014) noted that evidence of effectiveness of this specific training in reducing the likelihood of road-accident involvement is controversial, with some studies confirming the expectation and others failing to demonstrate the efficacy of training. However, there is broad agreement in the literature that training on hazard perception is a crucial aspect for safe driving. Other kinds of training might include the use of driving or riding simulators that, to our knowledge, is compulsory only in Japan (Haworth et al., 2005a). These types of training can be labeled as “active" because of the direct involvement of the participants, which makes the training more similar to real road conditions.

Motorcyclists are particularly vulnerable because they are more prone to more serious injury in the event of a crash compared to car drivers. In Italy, as the National Institute of Statistics (ISTAT) shows, despite the overall reduction in fatal crashes recorded in the 3-year period of 2010–2013, an enhancement from 0.72 to 0.84 in the mortality index (the ratio between the number of road user deaths and the number of vehicles involved in accidents ^∗^100) was recorded for moped vehicles in 2013 and, concerning motorcycles, the mortality index decreased but still remains the highest compared to the other vehicles (1.78 in 2012 and 1.68 in 2014; Istituto Nazionale di Statistica [ISTAT], 2014). The same ISTAT document stresses that 48% of fatal accidents involve two-wheeled vehicles, bicycles, and pedestrians, thus leading to the rating of two-wheeled vehicles as vulnerable vehicles also in the Italian context.

This greater vulnerability is linked both to the fact that two-wheelers are not protected by the bodywork and to the greater impact that some characteristics of the roads (such as holes in the road surface and, in general, covering conditions) might have on motorcyclists (Haworth et al., 2005b). For these reasons, the authors argued that the same aforementioned RT tasks (based on the passive viewing of video clips) employed to train hazard perception in car drivers might not be so effective for motorcycles because the way in which the behavior needs to be modulated after the hazard has been detected plays a more crucial role than simply pressing a button (i.e., responding as quickly as possible). Given the greater vulnerability of motorcycles, hazard-perception training seems even more crucial for motorcyclists than car drivers (Haworth et al., 2005b).

Moreover, it is worth noting that passive viewing of video clips, despite being the most common method used to train hazard perception, is a rather different condition with respect to road situations, in which we have to move in an environment where other road users are moving, thus adjusting our behavior to that of others. In these circumstances, we need to perform more complex actions than pressing a key so as to prevent hazards from becoming dangers. In addition, whereas in a typical RT paradigm the response is given after an explicit (conscious) recognition of the hazard, in more naturalistic situations hazard recognition must be performed at an implicit level while our consciousness is engaged in other tasks, such as remembering where we are going, reading the speed limit sign, and so on. Therefore, despite the fact that passive training for hazard perception is employed in license programs, it is evident that its efficacy on learning and transfer relies mainly on the level of involvement in the task, which in turn can be modulated through instructions and feedback delivery (Horswill, 2016).

In the last decade, efforts have been devoted to investigating how to train learner motorcyclists on hazard perception, both through traditional programs of road education including exposure to video clips of dangerous situations (passive training) and through the use of different kinds of simulators, which are considered “active training methods” and, for this reason, more similar to real road conditions (Wallace et al., 2005; Vidotto et al., 2011, 2015; Tagliabue et al., 2013). Thus, evidence has shown that both kinds of training are somewhat effective depending on the variables considered and on the aim of the study.

By comparing results from studies with simulators, real driving, and video-based exposition to hazardous scenarios focusing on perceptual-motor skills, other authors (see, for instance, Underwood et al., 2011; Crundall D. et al., 2013) concluded in favor of some amount of comparability between the tasks and exhorted that deeper investigations should be conducted on validity with reference to hazard perception.

Less attention has been paid to the comparison between methods in terms of the subprocesses involved. One key point that might explain inconsistencies in data derived from simulator and video-clip viewing is the quality of the feedback provided. As Horswill (2016) claimed, video-clip viewing techniques become more and more effective when feedback is delivered to the trainees and when they are allowed to determine for themselves the competency needed to correctly individuate indexes of potential hazards through the auto-production of running commentary, so as to improve awareness of potential hazards and one’s own ability to detect them. Concerning the simulators, the feedback is also delivered in terms of accidents or bad consequences of driving behavior, such as the need to make a hard brake, which prompts in the trainee emotional engagement; such emotional engagement, which can be supposed to contribute to the awareness of the potential hazards, has been demonstrated as being greater when compared to passive viewing of the same scenes (Tagliabue and Sarlo, 2015). This conclusion is in line with Horswill’s (2016) idea that passive experience has sometimes less of an impact on learning and leads us to focus on the subprocesses involved in learning through a riding simulator.

As a measure of emotional activation, Tagliabue and Sarlo (2015) used the skin conductance response (SCR), defined as a change in electrodermal activity developing in proximity of a risky or hazardous situation. The authors considered two characteristics of the SCR, i.e., amplitude, defined as the maximal increase in skin conductance in the window after the hazard scene relative to the baseline using a threshold of 0.05 μmho (cf. Boucsein et al., 2012; Kinnear et al., 2013), and the percentage of SCR (i.e., the proportion of SCRs detected over the total number of risky scenes). The results indicated that participants who were actively riding a moped simulator showed a higher percentage of SCRs compared to participants watching the same scenarios. Moreover, in scenes in which an accident occurred, the SCR amplitude was greater than in scenes without an accident.

These results confirmed and extended those of Kinnear et al. (2013), who demonstrated that during a hazard-perception test in which participants had to watch video clips of hazardous scenarios spotting incoming dangers, experienced drivers showed a greater percentage of SCRs than novice and learner drivers. The authors interpreted this result by noting that, in line with the idea of the existence of a dual modality system of risk appraisal (Slovic and Peters, 2006), experienced drivers are better at affective appraisal, thus providing a first contribution to the investigation of subprocessing involved in hazard perception.

Crundall (2016) stressed the need for research aimed to develop theoretical bases of hazard perception and its subcomponents, and for this reason, he investigated the ability to predict potential dangers on the basis of specific “precursors” present in different typical road conditions, considering hazard-prediction ability as the key point for hazard perception. Using a technique derived from the framework of *Situation Awareness*, the author investigated the predictive capability of different groups of road users. He showed that the performance in a task in which video clips of hazardous scenarios were stopped at the point at which a precursor of the impending hazard appeared was better in experienced drivers than in novice drivers, with experienced drivers showing higher accuracy in responding to three critical questions about the hazard: “What was the source of the hazard?”, “Where was the hazard located?” and “What happens next?” Interestingly, in Experiment 2, he varied the moment at which the video clips ended: in some clips, the last visible frame was the very first appearance of the precursor, e.g., the first moment in which the head of a pedestrian became visible, being his/her body masked from a parked car in proximity of a zebra crossing; in other clips the end was in a subsequent moment when the pedestrian was moving toward the zebra crossing; finally, the third kind of clip ended at a late moment when the pedestrian was completely visible and began to cross the street. The results showed that experienced drivers were more accurate than novices in all three end-point conditions, thus leading the author to conclude that the former are able to extract more information from early precursors (i.e., 1,250 ms before the actual occurrence of the hazard). Indeed, the ability to detect the precursors so early might reasonably be what allows experienced drivers to behave on the road so that dangers do not develop at all.

The strong emphasis placed on hazard perception led us to wonder whether there is any alternative measure of hazard perception that might provide more information about if and to what extent this ability can be improved. In a previous study by Di Stasi et al. (2011), in which ocular behavior was recorded while riding a Honda Riding Training (HRT) simulator, it was shown that experienced riders decrease their speed around locations in which hazards might occur more than first-time motorcyclists, and the authors interpreted this result in terms of the experts’ greater ability to anticipate risk. Moreover, by analyzing the number of accidents and the speed of the two groups, they concluded that novice riders are more prone to accidents not due to higher speed but rather because they are less conscious of the forthcoming danger. In another study, Crundall et al. (2003) compared the performance of three groups of participants (novice drivers, police drivers, and a control group matched for age and driving experience with the police group) watching video clips of hazardous scenes from the visual perspective of a police vehicle. The results revealed that police drivers and matched controls showed a shorter fixation duration and greater horizontal scanning as measured by the standard deviations of fixation locations. However, only police drivers showed a greater frequency of electrodermal responses, and the authors interpreted this result as an index of higher levels of hazard sensitivity in this group of participants in comparison with the others.

This aspect was directly investigated in Tagliabue and Sarlo’s (2015) previously mentioned study, in which the SCRs of a group of participants while riding a moped simulator were shown to be more frequent (in percentage) than that of a matched group of participants that had to press a key to detect hazards during the passive viewing of the same road situations; this result was interpreted in terms of a greater involvement in the simulator experience.

Thus, we reasoned that SCR studies might provide information about the mechanisms underlying the processes implied in hazard-perception training. This was the reason why we decided to focus on young adults with little on-road experience, hypothesizing that if simulator conditions really train the ability to anticipate hazards, then the implicit response – i.e., the SCR – should occur earlier when the participant re-experiences the same scene. Particularly, our aim was to bring out the mechanisms that might be crucial for the previously demonstrated (Di Stasi et al., 2011; Vidotto et al., 2011, 2015) reduction of accident rates in novice riders as experience with the simulator increases. In line with the idea that the reduction in accident rates depend on an improvement of the ability to detect hazards in advance and that SCR signals hazard detection (e.g., Tagliabue and Sarlo, 2015), we should predict earlier SCRs when participants perform the same task anew. The idea was that the ability to consciously detect the presence of a precursor and the SCR are, respectively, the cognitive and the implicit physiological/affective sides of the predictive component of hazard perception.

To the best of our knowledge, no previous studies have investigated the continuous changes in electrodermal activity while riding a moped simulator, by monitoring ongoing behavioral performance and skin conductance across a variety (i.e., 39, as explained in the Method section) of different hazardous scenarios. Moreover, the previously discussed studies measuring changes in electrodermal activity during passive viewing of video clips did not focus on the development of anticipation of psychophysiological responses when participants face again hazards on which they have been actively trained in a first session. Thus, the main innovative contribution of our work consists in the attempt to address these shortcomings.

The present study was the first step of the investigation aimed at casting light on the details of what develops during learning to avoid risk in terms of the mechanisms involved. First, on the basis of the considerations raised from studies that compared passive and active training methods, we decided to use the HRT simulator that has been demonstrated to provide greater involvement than other kinds of passive tasks. Second, we decided to test whether the improvement in performance during virtual riding with the HRT, which is well documented in the aforementioned studies, might be accounted for by speeding up the hazard-perception spotting, as proven by the anticipation of the psychophysiological response. The choice to focus only on inexperienced drivers/riders is then particularly crucial to be sure to “capture” the moment at which learning develops, being sure that it has not yet (fully) developed in the on-road experience.

Thus, we administered the same scenarios (called “courses” in the description of our methodology and procedure) to a group of young inexperienced drivers/riders in two different sessions, and, in line with the hypothesis that with HRT training participants learn to react more promptly to what Crundall (2016, p. 51) calls the “precursor of the impending hazard,” we expected that their electrodermal responses would occur earlier during the second administration of the same HRT courses than during the first.

## The Study

The aim of the present study was to investigate the psychophysiological mechanisms through which learning in hazard perception develops. The starting point relies on the results of previous research showing that (a) some groups of participants, professionally more involved in the kind of scenarios administered during hazard-perception training, showed greater electrodermal activity than other participants with a different level of driving experience or different professional experiences (Crundall et al., 2003); (b) experienced drivers showed more non-conscious responses to hazards, as measured by SCR percentages, than inexperienced drivers during the viewing of on-road hazardous video clips (Kinnear et al., 2013); (c) inexperienced riders showed SCRs both during the viewing of on-road scenarios and while actively riding through them, but psychophysiological reactivity was greater in the latter case (Tagliabue and Sarlo, 2015).

In the framework of the Somatic Marker Hypothesis (Damasio, 1994), to which Kinnear et al. (2013) explicitly referred, this implicit process should be anticipatory, but evidence on the anticipatory superiority of experienced drivers is not so clear in either Kinnear et al.’s (2013) or Crundall’s (2016) studies. We hypothesized that this failure could be due to the relatively low sensitivity of the methods employed, in that the video clips used for the test/training were divided into anticipatory and event periods or interrupted at different points before the danger happened, possibly interfering with the naturalistic development of participants’ responses. On these bases, we reasoned that a more naturalistic – even if virtual – procedure could have allowed us to capture the change in anticipatory capabilities of inexperienced drivers/riders trained with an HRT simulator.

Our prediction was that if learning to ride consists of an improvement in the ability to predict incoming dangers in advance so as to behave in such a way to prevent the real occurrence of dangers, and if this ability is indexed by increases in electrodermal activity, when the courses administered for the training are run again, we should record earlier SCRs along with a better performance.

### Method

#### Participants

Sixteen undergraduate students at the University of Padua who accepted to participate in the study were recruited. They included nine females and seven males between the ages of 19 and 24 (mean age 20). All participants were novice drivers/riders in that they had held their driver’s licenses for no more than 2.5 years (range 5–30 months; mean 12.3 months). Three students had a moped license for 4, 5 and 6 years, but had driven a car or moped for no more than 5,000 km overall (no one had license for motorcycles above 50 cc). Indeed, even though the study is focused on riding abilities, we also asked about car-driving habits so as to be sure that participants were really novice road users (this was crucial for our aims).

We set the inclusion criterion to 5,000 km of overall (with both cars and two-wheeled vehicles) road exposure considering the range of criteria used in the literature. Novice road users are defined as drivers with a mean annual mileage of less than 2,000 miles (3,218 km) and a mean driving experience of 2.9 years by Crundall et al. (2003), as riders that were either learner or licensed for no more than 12 months (Crundall D. et al., 2013) with a mean riding experience of 3,711 miles (5,972 km) per year (Crundall E. et al., 2013), or as drivers that had passed their driver’s license test no more than 3 years prior (Crundall, 2016) and that had driven a mean of 2,662 miles (4,284 km) in the last year (Kinnear et al., 2013). Our inclusion criteria are far below because they refer to overall road exposure with both two-wheeled vehicles and cars (not the mean mileage in the last year). This was decided to ensure that participants were truly inexperienced.

Participants were paid 13 euros per session for their participation in the experiment, had normal or correct-to-normal vision and were naïve to the purpose of the study. The study was conducted accordingly with the Helsinki declaration and received approval from the Ethical Committee for the Psychological Research of the University of Padua.

#### Apparatus and Stimuli

**FIGURE 1:**
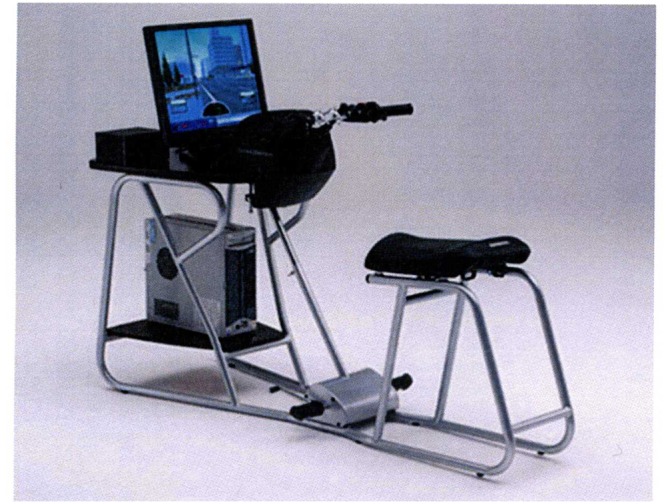
**The Honda Riding Trainer (HRT) simulator used in the present study**.

The HRT simulator (see **Figure 1**) consists of a Dell Pentium 4 pc (Windows^xp^ operative system) connected to a chassis with all the equipment of a motorcycle and to two screens: the first was the LCD monitor at a 1024 × 768 resolution on which the riding experience was delivered, and the second was placed on a table behind participants who were seated approximately 80 cm from the first monitor on a moped-like seat. The apparatus was set up as a moped (i.e., with an automatic transmission). The visual field covered a horizontal angle of 27.2° and a vertical angle of 21.7°. Two speakers that reproduced typical road noise and delivered instructions for navigation were also included in the apparatus.

**FIGURE 2:**
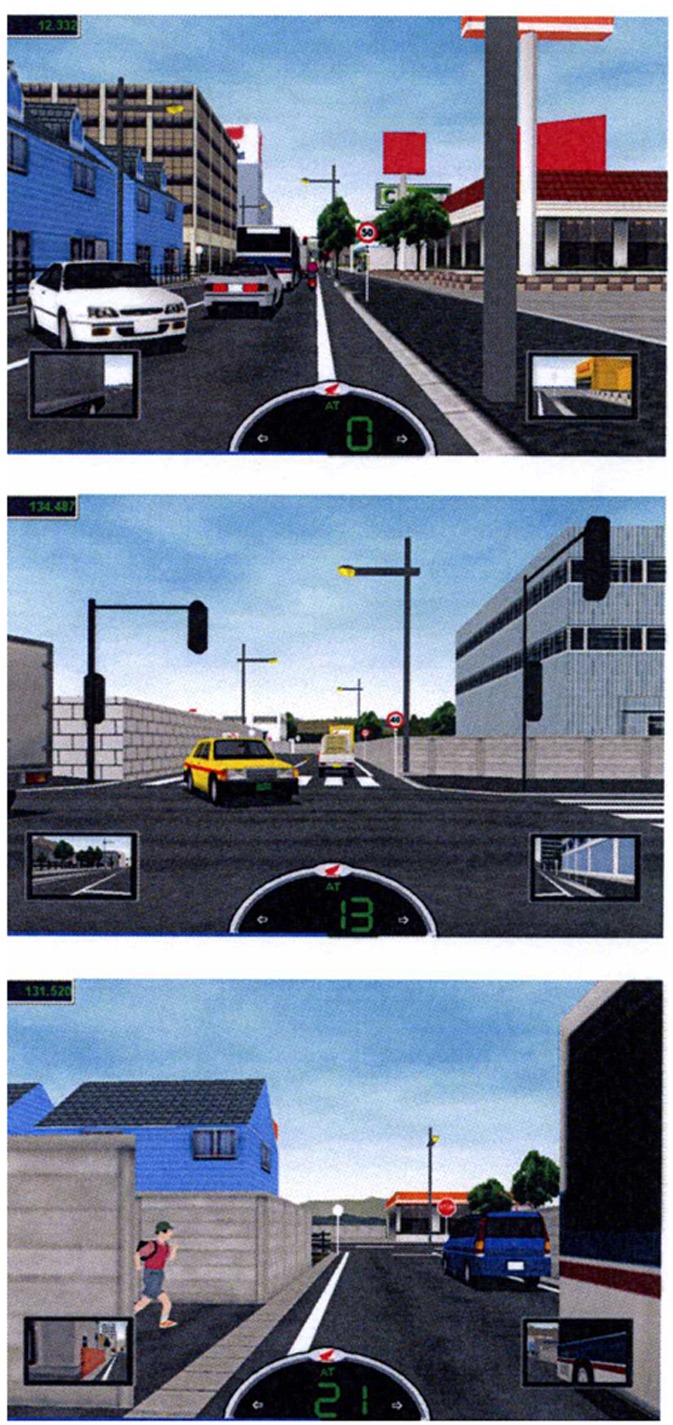
**Three frames depicting examples of the participants’ experience with the simulator.** For the description of all the scenes for each course, see the Appendix in the Supplementary Material.

The apparatus allowed us to show several road situations through which the participants had to ride and in which the most common risky scenes were represented, extracted from the report “In-depth investigation of motorcycle accidents” statistics (MAIDS, 2004). The different road situations were arranged in courses, and every course included 7 or 8 risky scenes that the rider had to face (see Appendix and **Figure 2**), for a total of 39 risky scenes.

Note that the HRT simulator is an interactive tool controlled by software generating dangerous scenes at the same position along each course for every participant. However, depending on the way in which participants modify their behavior (i.e., speed, position on the road, safe distance, and so on), the hazard they have to face may be more or less serious.

For the electrodermal activity recording, an amplifier system – a Grass Model SCA1 skin conductance coupler, associated with a Grass CP122 AC/DC Strain Gage amplifier (Grass Instrument Co., W. Warwick, RI, USA) – that returned the ongoing values of the electrodermal activity through a display, was placed near the second screen that reproduced what the participants were seeing while riding. A video camera recorded what was happening during the riding experience (shown by the second monitor) together with electrodermal activity (shown by the display of the amplifier) in an AVI file that was used during the coding phase.

#### Coding and Design

After the run of the experimental sessions, we extracted the .csv file provided by the simulator in which all the variables of the riding performance of participants were collected at a sampling rate of 30 Hz. Through the analysis of the AVI file obtained for each participant, we coded the values of electrodermal activity at the same sampling rate, so as to match the psychophysiological variable with the behavioral variables the simulator provided.

For each of the 39 hazard scenes in each session, we individuated the **clues**, i.e., the points, in terms of the position (*x*/*y* coordinates) of each participant along the path, after which hazards began to develop. For instance, if the hazard scene consisted of another motorcyclist beginning to cross an intersection from a lateral street without giving the right of way, the clue was the position of the participant when the first frame in which a part of the other motorcycle was visible. The clues were the same for every participant, and this allowed us to split all the scenes into two parts: *baseline*, a 5-s time window before the start of the hazard scene, providing the baseline for the electrodermal activity of each participant at that point of the course, and *post-clue*, a 10-s time window during which the hazard developed. The reason for choosing a 10-s time window was to ensure that the recorded increase in electrodermal activity was linked to the specific hazard, with no overlapping with other physiological changes due to other events present in the complex scenarios. This was particularly crucial because, in the present work, we were interested in measuring the onset anticipation of SCRs.

This onset anticipation was not measured in time units but in terms of distance covered after the clue. Thus, if in a certain section the participant was moving along the *x*-dimension, the onset anticipation was calculated in terms of the absolute difference (i.e., irrespectively from the left/right direction of the motion) between the *x*-value of the position of the participant when the clue appeared in the scenario and the *x*-value of the position of the participant when an SCR occurred. The same procedure was used for the y coordinate when the motion was in the up/down direction. Each unitary change in the *x* or in the *y* direction corresponds to a shift of approximately 1 m in the virtual scenario.

In summary, we calculated (for each participant and for each scene) the mean baseline value of the electrodermal activity and identified, in the post-clue time window, the moment at which an SCR, defined as an increase from baseline in electrodermal activity greater than 0.05 μmho, occurred (we chose the same threshold as in Kinnear et al., 2013 and in Tagliabue and Sarlo, 2015). After having identified all SCRs, we calculated the percentage of SCRs, i.e., the number of SCRs × 100/total number of the hazardous scenes in each course and session. In addition, the SCR onset was computed as the distance the participants covered after the clue until the appearance of the SCR. With regard to behavioral performance, we calculated the percentage of accidents as the number of accidents × 100/total number of the hazardous scenes in each course and session.

Overall, the design was a 2 (session) × 5 (course) repeated-measure design. The dependent variables were the percentage of accidents, percentage of SCRs and mean onset of SCRs.

#### Procedure

The participants filled out a questionnaire in which they provided data about age and driving and riding habits, and signed an informed consent form in which the basic characteristics of the procedure were described and the right to withdraw at any time was explicitly mentioned.

Then, participants were invited to sit on the moped seat, and two electrodes were placed with K-Y lubricating jelly on the left foot over the abductor hallucis muscle—adjacent to the sole of the foot and midway between the proximal phalanx of the big toe and a point directly beneath the ankle (see Boucsein et al., 2012). Instructions explained that participants had to ride following the vocal advice, complying with traffic laws and avoiding hazards and accidents. Finally, they were invited to inhale, hold and release their breath for a few seconds to test the reliability of the skin conductance recording.

The experiment consisted of two sessions scheduled a week apart. All participants were administered five courses per session representing peripheral roads at the HRT. As already noted, because each course might include 7 or 8 hazardous scenes (depending on the course; see Appendix), each participant faced a total of 39 potentially hazardous scenes in each session. The courses were administered according to degree of difficulty (derived from Miceli et al., 2008; Settanni, 2008) from the easiest to the most difficult. In the second session, the same five courses were administered in the same order so as to observe the expected anticipation in psychophysiological responses. Two brief courses without other road users were provided at the beginning of the first session to practice with the simulator. At the end of each course, a 3-min rest was allowed so as to bring back the skin conductance at the baseline level.

In each session, 39 potentially hazardous scenes were shown, and the participant had to ride in the virtual courses following vocal instructions indicating which turn he or she should take. Each session lasted approximately 45–60 min.

### Results

Analyses were performed with IBM SPSS Statistics 22. First of all, we carried out an ANOVA on the accident percentages so as to check for learning in terms of reduced likelihood of accidents. The ANOVA had one between-participants factor, i.e., *Gender*, and two within-participants factors, i.e., *Session* (two levels) and *Course* (five levels; from the first administered to the last). The *Gender* factor was added to control also for possible effects of gender in learning processes.

**FIGURE 3:**
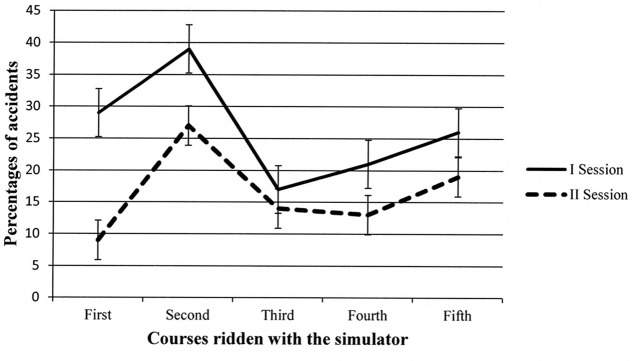
**Participants’ percentages of accidents in the different courses administered during the first and second session.** Error bars represent standard errors.

Two sources of variance reached significance: *Session*, *F*(1,14) = 31.64, *p* < 0.001, MSe = 132.85, and *Course*, *F*(4,56) = 6.19, *p* < 0.001, MSe = 243.22. The *Course* × *Gender* interaction was also significant, *F*(4,56) = 2.69, *p* < 0.05, MSe = 243.22. The *Gender* factor failed to reach significance (*p* = 0.06). As regards the *Session* factor, overall, the percentage of accidents was higher in the first than in the second session (Ms = 26% vs. 16%, respectively). Concerning the *Course* factor, as can be seen in **Figure 3**, after an increase from the first (i.e., the easiest) to the second course (*p* < 0.01 at the *post hoc* comparisons), percentage of accidents decreased in the third course (*p* < 0.01) and remained stable in the last two courses administered.

**FIGURE 4:**
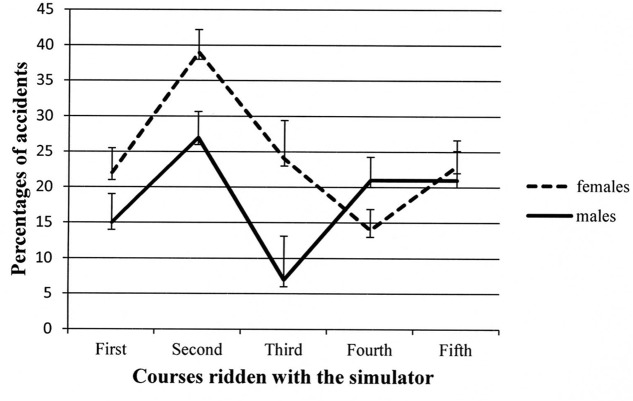
**Two-way interaction showing participants’ percentages of accidents for males and females in the different courses administered.** Error bars represent standard errors.

The *Course* ×*Gender* interaction is depicted in **Figure 4**. The trends of the two groups of participants were quite similar. The only significant differences in the *post hoc* tests were in the second and third courses, with males incurring fewer accidents than females. However, in the last two courses, the percentage of accidents was comparable in the two groups.

**FIGURE 5:**
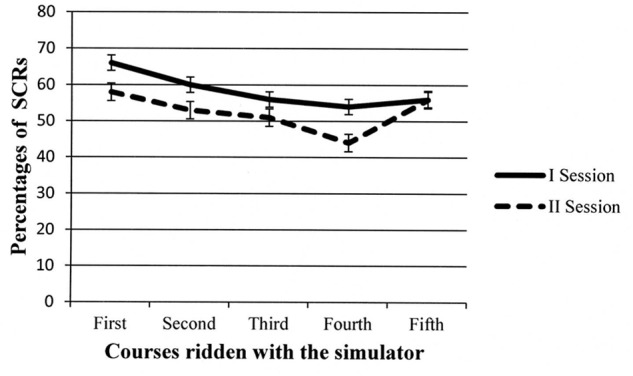
**Participants’ percentages of skin conductance response (SCRs) in the different courses administered during the first and second session.** Error bars represent standard errors.

A second ANOVA was conducted on the SCR percentages with the same design. Again, *Course* and the *Course* ×*Gender* interaction reached significance, *F*(4,56) = 2.93, *p* < 0.05, MSe = 247.07, and *F*(4,56) = 2.67, *p* < 0.05, MSe = 247.07, respectively. As can be seen in **Figure 5**, the percentage of SCRs decreased along the courses, and *post hoc* comparisons revealed that the SCR percentages of the third and fourth courses were different relative to the SCR percentage of the first course.

**FIGURE 6:**
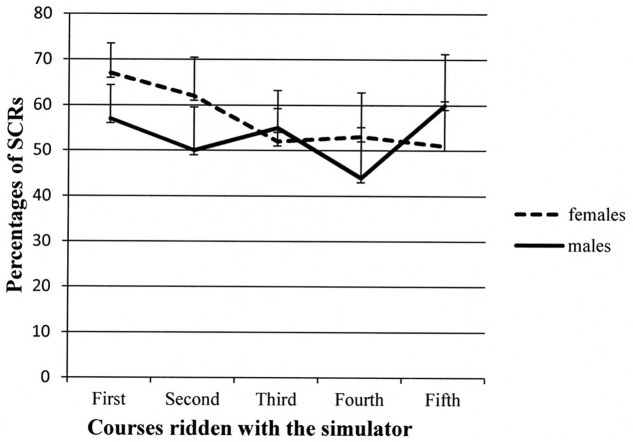
**Two-way interaction showing participants’ percentages of SCR for males and females in the different courses administered.** Error bars represent standard errors.

The *Course* ×*Gender* interaction is depicted in **Figure 6**, but *post hoc* tests failed in revealing significant comparisons. The trend seems to show that females showed higher SCR percentages in the first two courses, but in the last three courses, mean percentages of SCR were comparable to those shown by males, especially considering the wide variability attested by error bars. The *Gender* main effect was not significant (*p* = 0.73), whereas the *Session* main effect was marginally significant, *F*(1,14) = 3.57, *p* = 0.08, MSe = 405.33: the SCR percentages tended to decrease in the second session (Ms = 58% vs. 52%).

Finally, we conducted the same ANOVA as before on the SCR onset. Only the factor *Session* reached significance, *F*(1,11) = 6.85, *p* < 0.05, MSe = 43.51. However, in this analysis, three participants were discarded by the statistical program because of missing data, in that they had no SCRs in some level of the factor *Course* and this made the onset of the SCR incalculable. Note that this kind of missing data is not due to errors in the procedure or failures in data collection but is strictly related to the phenomena under investigation: if in some course no increase in electrodermal activity occurs, then, in that course, no SCR onset can be calculated.

Thus, we repeated the ANOVA without the *Course* factor (i.e., averaging participants’ SCR onset values across courses within each session). At this point, neither the factor *Session* factor nor the *Session* ×*Gender* interaction reached significance. To better understand the reason for such a difference, we inspected the data to understand whether some problem in data distribution might have been responsible for overturning previous results.

**FIGURE 7:**
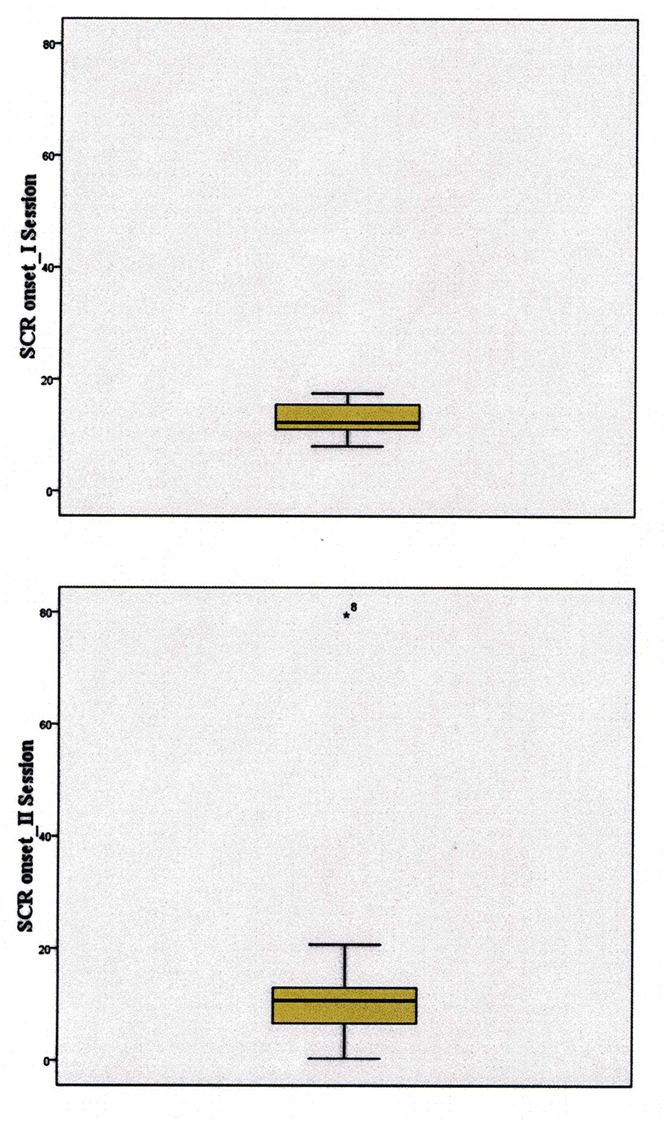
**The box plots of the distribution of data of the first (top panel) and second (bottom panel) session, indicating the outlier eliminated from the final analysis**.

As can be seen in **Figure 7**, the box plot of the distribution of the second session clearly showed the presence of one outlier. Thus, we repeated the ANOVA, discarding the outlier participant, and the significance of the *Session* factor replicated the results of the first ANOVA conducted on mean SCR onset, *F*(1,13) = 7.41, *p* < 0.05, MSe = 10.64. The mean SCR onset corresponded to 13 m in the first session and 10 m in the second session, thus showing clear anticipation. No other sources of variance reached significance.

It is worth noting that in our procedure we have used the courses and sessions to train as well as to assess the participants, and this might raise concerns. This is because we needed a tool that had good test–retest reliability, but, at the same time, that allowed to show improvement between sessions. In the literature, the reliability of SCR, as measured by test–retest correlation, was found to be moderate but significant during most baseline and test procedure periods (see Waters et al., 1987). Thus, to deepen this aspect, we carried out test–retest correlations on the dependent variables employed. The results showed significant Pearson correlations for all the dependent variables, with *R* varying from 0.53 for accident percentage (*p* < 0.05) and 0.54 for SCR onset (*p* < 0.05), to 0.87 for SCR percentage (*p* < 0.001). These results are consistent with the ANOVA results since *R*-values, although always significant, were lower when learning effects between sessions appeared.

### Discussion

The present data show that learning is evident in the second session, during which a stable decrease in percentages of accidents occurs. Considering progress in learning from the first to the last course within each session, the trend is not linear. This result is understandable considering that, in this trend, the effects of learning might be masked by the effects of task difficulty. Indeed, participants seemed to begin to ride cautiously in the first course, but then, in the second course, their percentage of accidents increased. However, as the training progressed, the number of accidents significantly decreased despite the progressive increase in task difficulty, proving that learning was being established. Therefore, our participants seemed to learn to ride better so as to avoid accidents. Even though this trend was present in both sessions (since the interaction *Session* ×*Course* was not significant), the percentage of accidents in the second session was significantly lower, thus confirming achievement of learning.

Concerning the differences between males and females, our results suggest different timings for learning. However, larger samples are needed to further investigate gender differences in hazard-perception training.

When considering the percentage of SCRs, the data indicate a decrease in the number of SCRs as training proceeded. This result requires deep consideration because, at first sight, it might appear counterintuitive. Indeed, considering the framework of hazard perception, one might argue that if learning implies becoming more able to detect hazards, then we should expect more frequent SCRs (greater sensitivity for hazards). As mentioned in the introduction, Crundall et al. (2003) recorded a greater frequency of electrodermal responses in police drivers, possibly due to higher levels of hazard sensitivity in this group of participants. However, this result was obtained in a passive detection task during which participants could not do anything to change drivers’ behavior in order to prevent the hazards. In contrast, in our study, participants were able to act in such a way so as to prevent the development of risky situations through progress in training. Thus, although the simulator presented the same potentially hazardous scenes at the same positions along the course (so that each clue was always linked to the same event), as participants’ ability to ride safely improved, less serious hazards might have occurred, and, consequently, less frequent changes in electrodermal activity might have been recorded.

In other words, given the simulator’s interactive nature, as a participant learns to drive in a safer way, he/she will be able to approach to the same scene in conditions that produce a lower degree of hazard. This is the key point of a safer riding style. Thus, as the rider becomes more and more prudent, reactions of his/her affective appraisal system are less often required. This should lead to a decrease in the number (and percentage) of changes in electrodermal activity. For instance, in the case of a child walking along the edge of the road who suddenly crosses the road when the rider approaches, if participants’ speed is high, the sudden change in the child’s behavior will trigger the activation of the appraisal system. As the rider learns to reduce his/her speed in proximity of children (having learned that they often behave unpredictably), the situation will be less hazardous, and the appraisal system might not be called to respond. In summary, learning to ride better means not only learning to avoid accidents when they are occurring but also to ride in such a way as to prevent a hazard from developing at all. If this was the case, obviously, fewer dangers should have been detected and, consequently, lower percentages of SCRs should have been expected.

Consequently, an improvement in riding abilities might lead to a greater capability to detect hazards and a greater ability to prevent the occurrence of such hazards. Unfortunately, these two complementary aspects of learning should lead to opposite results, as in the first case, we should expect an increase in the number of electrodermal responses, and in the second case, the occurrence of less serious hazards should lead to lower percentages of SCRs. This might be the reason for the marginal significance of the *Session* factor in the analysis of SCR percentage, as the greater ability of participants to drive so that hazards did not occur (which should have led to lower percentages of SCRs) might have masked their augmented sensitivity (which should have led to higher percentages of SCRs).

Taking all of these considerations into account, the results of the percentages of SCRs are in line with the idea that our participants improved their performance with the HRT, but the final response should be provided only by inspection of the readiness with which people react to hazards. That is, both in the case in which we have learned to ride so as to prevent the occurrence of hazards and in the case in which we have become more capable to detect them, learning should induce riders to react earlier. Indeed, our results of the onset anticipation of SCRs seem to confirm this important prediction: participants showed implicit activation in proximity of a hazard approximately 3 m before when they were faced with the same courses the second time.

## Limitations

The first limitation of the study is that the retest phase (our second session) was done with the same identical courses, but this was deliberately done to obtain the first-step result of what changed during learning, with all other conditions being equal. It can be argued that participants may have recalled some of the hazards from the first time riding through the course. Thus, the results observed in the retest phase (the second session) could partly reflect a memory effect. Indeed, this is what we expected, as we reasoned that, in line with Damasio’s Somatic Marker Hypothesis (Damasio, 1994), this is the way in which our appraisal system is supposed to work. As Damasio theorized, to develop anticipatory SCRs and, consequently, to learn avoiding hazards, re-exposure to the same situation is a necessary condition for the emotional system to predict decision outcomes under risk.

In other words, recall of prior experiences in the same situations is a prerequisite in learning to anticipate possible consequences of choice. This seems to be the way in which the somatic marker acts to modify our behavior: when the negative outcomes of our behavior are marked as unpleasant, the somatic marker reactivates when we encounter the same situation, thus acting as a reminder of the consequences expected. This would lead to the inhibition of the tendency to act in the same way or to the enhancement of the tendency to withdraw (for instance, in our conditions, it would inhibit the tendency to accelerate or enhance the tendency to decelerate).

This is what has been demonstrated to happen in the Iowa Gambling Task (Bechara et al., 1996), in which, after a certain number of disadvantageous choices, the memorized somatic responses that, at the beginning of the task, are recorded after the choice of the card, in the second part of the task are elicited before the potentially disadvantageous card is picked up to redirect the choice toward another, more advantageous deck. Thus, the first step of the way in which we learn through the appraisal system is based on the memories of our previous experiences. Our aim was to pinpoint this process. Only after having demonstrated that the somatic marker reactivates in the same scenes can we begin to consider generalization. If memories are effective in eliciting anticipatory somatic responses, than the generalization of the acquired learning to similar but different situations may be investigated. In other words, researchers wonder if simulator trainings provide learning that generalizes to real road conditions. Before reasoning about generalization, we would need to know what it is supposed to be generalized.

Another aspect that requires discussion concerns the sample composition, as it can be argued that because the majority of motorcyclists on the road are male, the sample employed might not be representative of the population. Regarding the rider population, recent evidence suggests that the use of mopeds and motorcycles is strongly increasing among females, and, consequently, the number of females killed or injured in two-wheeled vehicle crashes has increased (Organization for Economic Co-operation and Development [OECD]/International Transport Forum, 2015). The number of women who regularly ride a moped or a motorcycle is the highest over the last decades, especially in countries with high urbanization (Organization for Economic Co-operation and Development [OECD]/International Transport Forum, 2015). In the USA, the number of female riders more than doubled from 2003 to 2014 (Motorcycle Industry Council [MIC], 2015). This is especially true for the younger generations, in which more than 17% of motorcyclists are women (Motorcycle Industry Council [MIC], 2015). In the last decade, a similar situation has been recorded in Italy: 10.2% of females (vs. 19.7% of males) claim to use a moped or motorcycle (Associazione Nazionale fra le Imprese Assicuratrici [ANIA], 2009). To our knowledge, no statistics about the difference between males and females in the use of mopeds only are available, but it can be supposed that it might be also lower, considering the different characteristics of motorcycles and mopeds that make mopeds more easily maneuverable for women with regard to size and weight. For these reasons, we chose to balance, as much as possible, the number of males and females in our sample.

Gender differences in driving styles are controversial. By using self-report measures, some studies have reported more aggressive and risky driving behaviors in males than females (Dula and Ballard, 2003; Richer and Bergeron, 2012). However, questionnaires measuring “self-awareness” of a specific behavior, not necessarily reflect actual behavioral differences. Thus, we thought it was important to control also for possible effects of gender in the learning processes under investigation and our data indicate that, although some differences between genders seem present in the trends of learning, males and females reach the same level of learning during the simulator training.

Finally, concerning the limitations in the ecological validity of the present study, the alternative is to measure electrodermal activity in real on-road conditions. However, the nature of the electrophysiological variable recorded makes it difficult (though not impossible) to measure SCR in such conditions due to its sensitivity to movement artifacts, which are impossible to avoid on the road. For instance, Kinnear et al. (2013) cited “historical studies” dating back to between 1957 and 1978 that employed the same measure. In particular, both Taylor (1964) and Helander (1978) provided evidence in favor of the hypothesis that electrodermal response is related to traffic events in real on-road conditions. In addition, it was demonstrated that both acceleration and braking are preceded by SCR (Helander, 1978). However, in real traffic conditions, it is not so easy to create comparable situations for all participants because no control over the other road users is possible. On the other hand, simulator studies provide results that are much more comparable and detailed albeit less ecological. Surely, the convergence of evidence from simulators and real road conditions will allow the provision of more complete and useful information.

## Conclusion

The current study arose within the framework of investigations of the role of hazard perception in road safety. Particularly, we referred to what Slovic and Peters (2006) and Kinnear et al. (2013) called risk appraisal or affective appraisal, as measured by changes in electrodermal response, i.e., SCR. In a previous study, Tagliabue and Sarlo (2015) demonstrated that the percentage of SCRs was higher in scenes without accidents, and it was explained considering that avoiding accidents means to detect hazards through SCRs so as to play out emergency maneuvers to avoid a crash. This happens during the first stage of learning, which presumably consists of becoming more sensitive to hazards.

In the present study, we wanted to focus on how the implicit mechanism of hazard perception develops. For this purpose, we first replicated the experimental procedure of Tagliabue and Sarlo (2015) consisting of riding a virtual moped on road courses and then re-administered the same courses to understand how SCRs vary when participants face conditions they have already encountered. We reasoned that in more advanced stages of learning, an improvement in the ability to detect and avoid hazards should lead to safer behaviors with a consequent reduction in the occurrence of hazards and accidents but, crucially, with an anticipation of the SCR onset, which would attest to greater readiness in the implicit, unconscious and prompt detection of the presence of hazards. For this reason, we focused our analysis on the dynamic changes of SCRs, both through the different courses presented in each session and through the two different sessions.

The results showed a reduction in accidents with progress in training both within and between the sessions. This reduction in accidents was also accompanied by a reduction of developing hazards, as proven by the reduction in percentages of SCRs from the first to the fifth course. The fact that in the second session, the percentage of SCRs was only slightly lower than in the first session is in line with the idea that hazard-perception learning is a complex phenomenon that implies two complementary processes leading to opposite consequences: (a) a greater sensitivity to hazards and (b) a greater ability to behave so that the hazard does not occur at all.

The final confirmation for these arguments comes from the analysis of SCR-onset anticipation. The earlier mean SCR onset in the second session testifies that learning has been effective in making riders faster in their implicit response to hazard, and this should have granted them more time to avoid risks and accidents.

It is worth noting that the computation of SCR onset in terms of spatial distance of the beginning of the SCR from the clue allowed us to compare the physiological reactivity of participants independently from their speed and driving behavior to ensure that the recorded SCR was related to the same road events for all participants.

## Directions for Future Research

The present study demonstrated that in the virtual reality of the HRT simulator, we learn to detect the already-encountered hazard earlier through the electrodermal response elicited by the appraisal system. This represents the first stage of a three-step investigation aimed at understanding the implicit mechanisms on which learning of hazard anticipation relies. The following step will be to investigate if and how this learning generalizes to other virtual situations, similar to those already experienced in their basic structure (other courses including similar hazards) but different in specific features.

Moreover, the ultimate goal will be the comparison between the performance observed during the simulation with the on-road performance, which should be the final aim of longitudinal research designed to monitor the occurrence of real on-road accidents in participants trained with the simulator compared to other modalities of training over the years. However, as Horswill (2016) claimed, despite the fact that evidence in the literature does not yet completely confirm if simulator trainings (as well as other kinds of training methods) actually reduce on-road crash risk, understanding the behavioral measures that are affected by each kind of training is still important and can allow us to predict crash risk.

Despite the limitations just mentioned, some degree of validity of the simulator was attested to by Underwood et al. (2011) and Meuleners and Fraser (2015), who demonstrated the comparability of ocular behaviors, mirror-check behaviors, attention to traffic lights and stop signals, speed at intersections and speed maintenance observed in simulation sessions and during on-road experiences. As Goode et al. (2013) noted, the effectiveness of simulator trainings is enhanced when studies focus on higher-order cognitive abilities, among which hazard perception is absolutely included.

In summary, simulators might provide the opportunity to conduct fine-grained analyses of the underlying mechanisms involved. This is possible, at least in part, with video-clip techniques as well, but in this case, the development of mechanisms depending on the behavior played out by the road user is not observable. The understanding of the development across learning of the ongoing implicit responses of potential road users to incoming hazards might provide additional hints to analyze the effectiveness of the variety of methods employed in the field of road education.

## Author Contributions

MT conducted the data collection and coding, statistical analyses and manuscript writing. EG contributed to the data coding, statistical analyses, and results discussion. MS contributed to the results discussion and manuscript writing.

## Conflict of Interest Statement

The authors declare that the research was conducted in the absence of any commercial or financial relationships that could be construed as a potential conflict of interest.
