# Supplementary material for: A First Step toward the Understanding of Implicit Learning of Hazard Anticipation in Inexperienced Road Users Through a Moped-Riding Simulator
Source: Front Psychol. 2017 May 11;8:768. doi: 10.3389/fpsyg.2017.00768 (PMC5425582; doi:10.3389/fpsyg.2017.00768)
Supplement: Supplementary file 1 [file Data_Sheet_1.docx]

**APPENDIX**

List of the 39 hazard scenes faced by participants.

| COURSE 1 |  |
| --- | --- |
| Scene 1 | A motorcycle coming from a left road and hidden by a preceding car appears suddenly when participant is turning left paying attention to another car coming from the road ahead. |
| Scene 2 | A ball coming from a gate on the right invades the road, followed, shortly after, by a child. |
| Scene 3 | The participant has to entry in the main street turning left while a truck on the left hides the view. |
| Scene 4 | A child starts to cross the road on a pedestrian crossing. When the participant starts to move toward the lane just crossed by the child, he suddenly changes direction turning back. |
| Scene 5 | At a traffic-light intersection the participant starts to cross the intersection going ahead when a truck turns right cutting in front of him. |
| Scene 6 | The door of a car parked on the right is suddenly opened while participant is coming. |
| Scene 7 | A child crossing the road on a pedestrian crossing suddenly appears from behind a bus at the bus stop. |
| Scene 8 | A car coming from the opposite direction invades the lane of the participant. |
| COURSE 2 |  |
| Scene 1 | A truck traveling in the same direction of the participant turns right in a parking area, hiding the view. A car enters in the lane from the same parking area. |
| Scene 2 | The participant is traveling preceded by a small truck. The carriageway is narrowed due to an obstruction and when the participant moves toward the central line to avoid it, a car coming from the opposite direction suddenly appears close to the central line. |
| Scene 3 | At a traffic-light intersection with a green light, the participant starts to turn left but has to stop in the middle to allow a bus to turn right from the left road. Meanwhile the traffic light turns to red and a motorcycle coming from ahead of the intersection starts to move. |
| Scene 4 | A bicycle suddenly starts to move at the extreme right of the field of view through a pedestrian crossing when the driver turns right at a junction. |
| Scene 5 | A vehicle suddenly invades the lane of the driver in proximity to a traffic light junction. |
| Scene 6 | A vehicle coming from the road on the right, in which the participant has to turn, invades the lane just near the turn. |
| Scene 7 | A pedestrian suddenly crosses the road in a situation of overcrowding of the road, such as his view is hidden by other vehicles. |
| Scene 8 | A child, walking along the edge of the road in the same direction as the participant, moves far from the side of the road to pass a parked car. |
| COURSE 3 |  |
| Scene 1 | While starting a left turn at an intersection, a motorcycle suddenly appears behind a truck and enters the intersection. |
| Scene 2 | A vehicle parked at the edge of the road sudden starts to move while the participant is approaching it. |
| Scene 3 | A small truck starts to enter a parking area on the left and then makes a reverse invading the lane of the participant. |
| Scene 4 | At a traffic light a truck does not allow the driver to notice a taxi which is about to turn. |
| Scene 5 | A pedestrian hidden by a parked vehicle suddenly crosses the road. |
| Scene 6 | A vehicle traveling in front of the participant in the same direction suddenly stops. |
| Scene 7 | A vehicle enters from the right close to an intersection whose visibility is reduced by a parked truck. |
| Scene 8 | When the participant is entering into a main road, a bicycle suddenly crosses the road with no pedestrian crossing. |
| COURSE 4 |  |
| Scene 1 | A vehicle suddenly coms out from a parking area on the right while a pedestrian on the other side of the street catches the attention of the participant. |
| Scene 2 | The bicycle that precedes the driver suddenly shifts to the left to turn, cutting in front of the participant. |
| Scene 3 | While approaching to an intersection, participant can’t see the stop signal hidden by a parked truck risking to collide with a bicycle that is entering from the left. |
| Scene 4 | A child suddenly crosses the road. |
| Scene 5 | A pedestrian hidden by an oncoming vehicle suddenly crosses the road. |
| Scene 6 | A vehicle traveling ahead the participant suddenly makes a U-turn. |
| Scene 7 | Passing a rail crossing, a bicycle occupies the participant’s lane to avoid a pedestrian. |
| COURSE 5 |  |
| Scene 1 | The dense traffic situation does not allow the driver to notice a vehicle coming from the opposite lane that is about to turn crossing the road. |
| Scene 2 | A vehicle suddenly turns right to enter in a parking area, cutting in front of the participant. |
| Scene 3 | While crossing and intersection, the light turns yellow and the participant risks a collision with a vehicle coming from the opposite lane and turning left, cutting in front of him. |
| Scene 4 | Near a turn, a lane is closed for road works. Participant has to pass paying attention to vehicles coming from ahead with the turn hiding the view. |
| Scene 5 | A vehicle coming from the opposite direction occupies the participant’s lane during an overtaking. |
| Scene 6 | A pedestrian close to a parked vehicle suddenly starts to cross the road while the participants is coming. |
| Scene 7 | A pedestrian hidden by a truck in a queue suddenly crosses the road. |
| Scene 8 | After turning on a siren, an emergency vehicle starts to occupy the participant’s lane to overtake a stopping vehicle. |
